# A case of respiratory toxigenic diphtheria: contact tracing results and considerations following a 30-year disease-free interval, Catalonia, Spain, 2015

**DOI:** 10.2807/1560-7917.ES.2018.23.13.17-00183

**Published:** 2018-03-29

**Authors:** Mireia Jané, Maria José Vidal, Neus Camps, Magda Campins, Ana Martínez, Joan Balcells, Maria Teresa Martin-Gomez, Gloria Bassets, Silvia Herrera-León, Anton Foguet, Mar Maresma, Nuria Follia, Sonia Uriona, Tomàs Pumarola

**Affiliations:** 1 Public Health Agency of Catalonia, Barcelona and Girona, Spain; 2 CIBER Epidemiology and Public Health (CIBERESP), Carlos III Institute of Health, Madrid, Spain; 3 Vall d’Hebron University Hospital, Catalan Institute of Health, Barcelona, Spain; 4 Centre d’Atenció Primària d’Olot, Catalan Institute of Health, Olot, Spain; 5 National Microbiology Centre, Carlos III Institute of Health, Madrid, Spain; 6 Fundació Hospital d’Olot i Comarcal de la Garrotxa, Olot, Spain

**Keywords:** Diphtheria, outbreak, control measures, prevention, diphtheria antitoxin, public health, contact tracing

## Abstract

In May 2015, following a 30-year diphtheria-free interval in Catalonia, an unvaccinated 6-year-old child was diagnosed with diphtheria caused by toxigenic *Corynebacterium diphtheriae*. After a difficult search for equine-derived diphtheria antitoxin (DAT), the child received the DAT 4 days later but died at the end of June. Two hundred and seventeen contacts were identified in relation to the index case, and their vaccination statuses were analysed, updated and completed. Of these, 140 contacts underwent physical examination and throat swabs were taken from them for analysis. Results were positive for toxigenic *C. diphtheriae* in 10 contacts; nine were asymptomatic vaccinated children who had been in contact with the index case and one was a parent of one of the nine children. Active surveillance of the 217 contacts was initiated by healthcare workers from hospitals and primary healthcare centres, together with public health epidemiological support. Lack of availability of DAT was an issue in our case. Such lack could be circumvented by the implementation of an international fast-track procedure to obtain it in a timely manner. Maintaining primary vaccination coverage for children and increasing booster-dose immunisation against diphtheria in the adult population is of key importance.

## Background

Diphtheria, a vaccine-preventable disease, is an acute bacterial infection that affects mainly the upper respiratory tract and, less frequently, the skin. It is caused by the action of a diphtheria toxin which is produced by toxigenic *Corynebacterium diphtheriae*, *C. ulcerans or C. pseudotuberculosis.* The toxin-producing *C. diphtheriae* is transmitted via respiratory droplets by close contact. The incubation period for *C. diphtheriae* ranges from 2 to 7 days, but can be as long as 10 days. No significant reservoirs for *C. diphtheriae* other than humans have been identified [1,2].

An overview of the reported number of cases of the three main types of diphtheria in Europe shows that they remain low, but circulation continues in some European countries, including Latvia, Russia and Ukraine which have had outbreaks in the past [3,4]. Case reports from the United Kingdom (UK) [5-8], France [9], Belgium [10], Sweden [11], Finland [12], Norway [13] and Germany [14] have been published in recent years. Further reports show an increase in the number of reported diphtheria cases in the European Union (EU), mainly from people travelling to and from endemic areas [15,16]. In the EU/European Economic Area (EEA) from 2009 to 2014, there was an increase of 280% in the number of cases, reaching 38 cases in 2014 [10,16]. In Spain, the last two cases of diphtheria were diagnosed in 1986 [17].

Globalisation implies increased travelling and continuous migration flows, the latter often related to wars and natural catastrophes [18]. In such situations, vaccine preventable diseases may spread from areas where they are still endemic and vaccinations, which have been shown to be a very important public health achievement, need to be reinforced. In Europe, vaccination has greatly reduced many infectious diseases, including diphtheria [19,20].

Catalonia, a region in the north-east of Spain, has an effective immunisation programme. The routine vaccination scheme includes primary immunisation against diphtheria, tetanus and acellular pertussis with the DTPa vaccine at the ages of 2, 4, 6 and 18 months, with subsequent booster doses for diphteria received at 6 and 14 years of age via the tetanus–diphtheria (Td) vaccine. Then, at 40 and 65 years of age, another booster of Td is recommended [21,22]. The primary vaccination coverage against diphtheria in children younger than one year of age in Catalonia reached 94% in 2016 [23]. However, some groups of the population are refraining from vaccination for personal beliefs or religious reasons. Studies have explored the reasons for parental refusal in order to be able to improve information given to parents related to the benefits of vaccination [24,25].

### Outbreak detection

On 25 May 2015 (day 2), an unvaccinated 6-year-old child was taken to their primary healthcare physician with a 48-hour history of sore throat and fever. The child was prescribed amoxicillin. On 28 May, the child was taken to a local hospital emergency unit with a worsening condition after 5 days of general malaise, fever, persistent sore throat, inability to swallow and an increase in neck diameter. The physical examination showed hypertrophic tonsils with easily bleeding pseudo-membranous plaques and necrotic areas. The child was diagnosed as a suspected case of diphtheria, admitted to an isolation ward and treated with penicillin G sodium, piperacillin/tazobactam and vancomycin intravenously. The case was reported to the Epidemiology Surveillance and Rapid Public Health Response Department at the Public Health Agency of Catalonia and then to the Spanish Alert and Emergencies Coordination Network that communicated the case to the European Centre for Disease Prevention and Control (ECDC).

On 29 May (day 6), a throat swab was sent to the Spanish National Centre for Microbiology. On 30 May (day 7), a positive PCR showing the presence of a *C. diphtheriae* *tox* gene was obtained. The genotype of *C. diphtheriae* was determined using multilocus sequence typing (MLST) as described by Bolt et al. [26]. The isolated genotype belonged to the sequence type ST-377. On 5 June, an Elek test carried out by the World Health Organization Collaborating Laboratory for Diphtheria and Streptococcal Infections at Public Health England, Colindale, UK, confirmed the expression of the toxin.

On 31 May (day 8), the patient was transferred to a paediatric intensive care unit due to a progressive worsening of their clinical condition. The Public Health Agency of Catalonia, in coordination with the Spanish Alert and Emergencies Coordination Network and the Spanish Agency of Medicines and Medical Devices (AEMPS), started searching for equine-derived diphtheria antitoxin (DAT), which lacked availability in most European countries [27]. The DAT was eventually made available through international collaboration and was obtained from France and Russia. On 1 June (day 9), an initial dose of DAT (120,000 IU) [2,28,29] produced by the Institute of Immunology Zagreb, Croatia, was sent to Catalonia from France and administered to the child. A second dose (50,000 IU) was administered 12 hours later and a third dose (70,000 IU) on 3 June (day 11), both obtained from Russia. Unfortunately, after being in a critical condition and on extracorporeal life support for several days, the patient died on 27 June because of myocardial dysfunction and neurological complications.

This article aims to describe the epidemiological and microbiological investigation, as well as public health coordination in order to highlight some learned lessons and implications from the experience.

## Methods

### Epidemiological investigation

The epidemiological investigation was carried out by the Public Health Agency of Catalonia in collaboration with the local healthcare network. The epidemiological investigation and contact monitoring was based on the Catalan protocol for action against a case of diphtheria [28]. According to the protocol, the confirmation of one case of diphtheria is considered an outbreak, for the purpose of the investigation and the application of control measures. A confirmed case of respiratory toxigenic diphtheria is defined as a person with upper respiratory illness characterised by sore throat, fever and an adherent membrane of the tonsils, pharynx or nose, together with laboratory confirmation. A probable case is defined as a person with classic respiratory diphtheria and an epidemiological link with a confirmed case, human or animal. A suspected case is defined as a person with clinical symptoms of respiratory toxigenic diphtheria. An asymptomatic carrier of respiratory toxigenic diphtheria is defined as a patient with no symptoms but with laboratory confirmation of toxigenic *C. diphtheriae.* A positive PCR that shows the presence of the *tox* gene in the clinical samples of *C. diphtheriae, C. ulcerans o*r *C. pseudotuberculosis* needs the expression of the toxin by the Elek test to be laboratory confirmed.

The 6-year-old index case lived with their family in a city in the north-east of Catalonia. The child and a younger sibling did not receive any vaccines because of parental decision. The child did not have a travel history and had not been in contact with individuals who had recently been in a country with endemic transmission. The child attended a school-associated camp with 57 schoolmates the week before the onset of symptoms.

Immediately after the suspected case was reported to the Public Health Agency of Catalonia on 28 May (day 5), an investigation was conducted by the epidemiological team at the Agency to implement prevention and control measures according to local protocol for the management of a case of diphtheria [28]. An epidemiological team interviewed the physician who reported the case and contacted the child’s family, the school management team and healthcare professionals who attended the index case. People who had been in contact with the index case were identified by public health epidemiologists, primary healthcare physicians and nurses. Teachers, tutors and all parents with children who shared curricular and extracurricular activities after school were contacted and advised to go to their primary healthcare centre to review the vaccination status of their children and have their children’s throat swabbed for analysis of toxigenic *C. diphtheriae*.

To compliment that, the epidemiological team and a paediatrician went to the school to inform all parents, answer their questions and get a throat swab for analysis from all the children who had been in close contact with the index case.

### Microbiological investigation

In the first stage of investigation, 178 contacts underwent physical examination looking particularly for the presence of classic respiratory symptoms and fever. Throat swab samples collected at healthcare centres and the school were sent to the National Centre for Microbiology for analysis by PCR. Nine of the 178 swabs were positive for toxigenic *C. diphtheriae*, with all nine taken from asymptomatic vaccinated children who had been sharing a room with the index case during the school-associated camp. Because of this finding, a second contact investigation began, with 39 family members of these nine positive children being identified and having throats swabs taken for study. This second stage resulted in one positive sample, which came from a fully vaccinated parent of one of the nine children.

## Results

### Close contacts and contact tracing

Contact tracing occurred in two stages, with a total of 217 contacts being identified. The first stage arising via the epidemiological investigation detailed above, identified the following contacts: family members of the index case (n = 7), camp tutors, schoolmates and teachers (n = 94), schoolmates and tutors from a swimming pool outside school (n = 27) and healthcare workers in contact with the case before isolation (n = 50). The second stage identified family members (n = 39) of 9 children found to be carriers. The vaccination status of all contacts was checked. In total, 114 contacts had had their vaccination completed in the previous 5 years, while for the remaining 103, it was incomplete or unknown. Therefore, all 103 received a booster dose and had their immunisation status completed (Figure).

**Figure:**
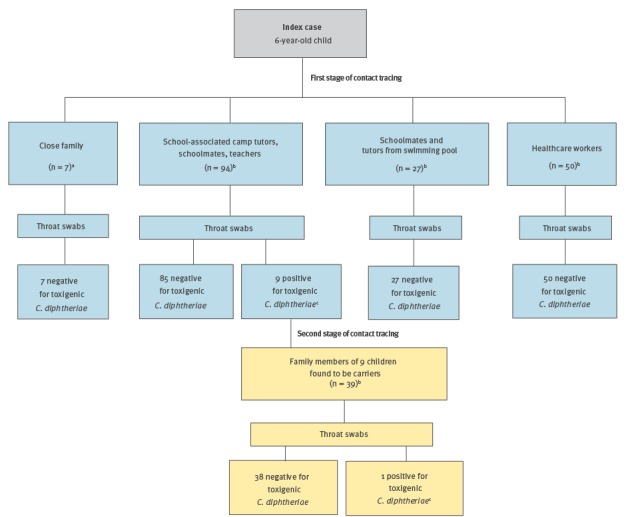
Index case of diphtheria’s contacts identified via two stages of an epidemiological investigation, Catalonia, Spain, 2015

^a^ Prophylactic antibiotic treatment and a booster dose of diphtheria vaccination (adults)/primary immunisation dose (unvaccinated younger sibling).

^b^ Some of these individuals received a booster dose of diphtheria vaccination and had their vaccination status updated.

^c^ Antibiotic treatment. All 10 carriers were isolated at home until they had two negative throat swabs; one obtained 24 hours after completing the treatment and one obtained 24 hours after the first negative throat swab.

### Microbiological investigation

At the end of the investigation, 10 carriers, nine children and one adult, had been confirmed. *C. diphtheriae* strains were identified in all carriers. All the isolates were characterised by MLST [26] and all genetic lineages belonged to sequence types ST-377, which was that of the index case isolate.

### Outbreak control measures

The child’s family immediately received the prophylactic antibiotic treatment and throat swabs for analysis were taken. Both parents and three grandparents were given an intramuscular dose of benzathine penicillin (1,200,000 IU). The fourth grandparent, who was allergic to penicillin, was given erythromycin (1 g orally daily for 10 days). The younger sibling of the index case was given an intramuscular dose of benzathine penicillin (600,000 IU). All six adults were also given a booster dose of diphtheria vaccine. The younger sibling had never been vaccinated, and received a primary immunisation dose according to recommended vaccination schedule [21].

All carriers were given the antibiotic treatment according to regional, national and international recommendations [2,28-30]. Children younger than 6 years of age received an intramuscular dose of benzathine penicillin of 600,000 IU as soon as they were identified as a carrier, while individuals 6 years of age and above received a dose of 1,200,000 IU. After a week, two carriers had negative throat swabs while eight remained positive for *C. diphtheriae.* As a result of this, these eight carriers also received oral erythromycin (40 mg/kg/day for children and 1 g/day for the adult) for 10 days. In addition, all carriers were isolated at home until they had two negative throat swabs; one obtained 24 hours after completing the treatment and one obtained 24 hours after the first negative throat swab.

During the investigation, there was enhanced surveillance by healthcare professionals and public health epidemiologists. The Public Health Agency of Catalonia sent the protocol to all healthcare centres, reminding them of the situation and the need to notify any suspected case. Once samples from the index case’s schoolmates were found positive for diphtheria, an advisory committee that included epidemiologists, paediatricians, microbiologists, internal medicine specialists, general practitioners and pharmacologists was established by the Public Health Agency of Catalonia. Its purpose was to offer support, advice and recommendations on the management of the epidemiological investigation. Recommendations were prepared for all Catalan families in order to promote the review of the vaccination statuses of their children and to make sure they were up-to-date before sending them to summer camps. A risk communication plan was created to regularly inform the media and avoid public alarm.

## Discussion and lessons learnt

After being diphtheria-free for more than 30 years, a confirmed case was found in Catalonia at the end of May 2015 [1]. Once the physician reported the suspected case, a public health emergency team began an urgent coordination among various stakeholders. Coordinated efforts and collaborative actions between the public health surveillance system and other healthcare partners such as local and teaching hospitals, primary healthcare centres, emergency medical services, and health and educational authorities were absolutely essential to control the outbreak and should be maintained and reinforced in order to detect a suspected future case of diphtheria as quickly as possible.

The fact that the investigation found 10 vaccinated carriers who did not develop the disease shows that efforts should focus on maintaining primary immunization coverage and improving the booster dose coverage in under-vaccinated populations to prevent serious illness.

This fatal case of an unvaccinated 6-year-old child shows that maintaining high vaccination coverage of diphtheria vaccination among the population is essential to prevent introduction of the disease from areas where *C. diphtheria* still circulates. It is thus necessary to increase health education and promote the importance of childhood vaccination to all paediatricians and everyone working within public health and education.

This case also highlights the need for rapid treatment with DAT in combination with antibiotics. Early administration of such is essential to achieving a good outcome for the patient. One of the most critical issues was the shortage of DAT for immediate use when clinicians suspected diphtheria. The shortage of DAT in the EU, which has also been acknowledged by others [31], made it very difficult to obtain it. Given that another fatal case was reported in Belgium in 2016 [10] and an imported case of toxigenic cutaneous diphtheria was diagnosed in Catalonia in summer 2016, there is clearly an urgent need for more rapid access to DAT in Europe [27,31]. It would be helpful to organise and have a stockpile for all EU countries, and to maintain an inventory of DAT availability within and between countries [27]. Ensuring that all health authorities know the formal procedures to rapidly obtain DAT and having adequate access to diphtheria therapy should be a crucial goal. Another possible strategy to overcome the lack of DAT availability could involve using plasma obtained from young adults receiving a booster dose of diphtheria vaccine [32].

While the objective of the investigation was not to find the source of the infection, the family with two carriers, a child and an adult, might have been the source although this could not be confirmed. These two individuals did not have a travel history but had been in contact with individuals from countries in Europe with circulation of *C.* *diphtheriae*. As far as it was known, these individuals did not show any symptoms compatible with diphteria.

## Conclusions

The reported outbreak is a reminder that in non-endemic countries with high vaccination coverage, people who are not vaccinated against diphtheria are at a risk of developing clinical illness because vaccinated people may carry *C.* *diphtheriae* and be asymptomatic. Therefore, health and public health professionals should be aware of this situation and reinforce coordinated epidemiological surveillance efforts. The lack of DAT availability is a challenge for necessary timely measures that should be overcome.
